# Negative frequency‐dependent selection maintains shell banding polymorphisms in two marine snails (*Littorina fabalis* and *Littorina saxatilis*)

**DOI:** 10.1002/ece3.7489

**Published:** 2021-05-01

**Authors:** Daniel Estévez, Juan Galindo, Emilio Rolán‐Alvarez

**Affiliations:** ^1^ Departamento de Bioquímica Genética e Inmunología y Centro de Investigación Mariña (CIM‐UVIGO) Universidade de Vigo Vigo Spain; ^2^ Greenland Institute of Natural Resources Department of Fish and Shellfish Nuuk Greenland

**Keywords:** apostatic selection, camouflage, color polymorphism, frequency‐dependent natural selection, genetic drift, *Littorina*

## Abstract

The presence of shell bands is common in gastropods. Both the marine snails *Littorina fabalis* and *Lttorina saxatilis* are polymorphic for this trait. Such polymorphism would be expected to be lost by the action of genetic drift or directional selection, but it appears to be widespread at relatively constant frequencies. This suggests it is maintained by balancing selection on the trait or on a genetically linked trait. Using long time series of empirical data, we compared potential effects of genetic drift and negative frequency‐dependent selection (NFDS) in the two species. The contribution of genetic drift to changes in the frequency of bands in *L. fabalis* was estimated using the effective population size estimated from microsatellite data, while the effect of genetic drift in *L. saxatilis* was derived from previously published study. Frequency‐dependent selection was assessed by comparing the cross‐product estimator of fitness with the frequency of the polymorphism across years using a regression analysis. Both studied species showed patterns of NFDS. In addition, in *L. fabalis*, contributions from genetic drift could explain some of the changes in banding frequency. Overdominance and heterogeneous selection did not fit well to our data. The possible biological explanations resulting in the maintenance of the banding polymorphism are discussed.

## Graphical abstract

Invertebrate shell polymorphisms are widespread. We studied the maintenance of the polymorphism of shell bands in two species of littorinids. We found evidence of negative frequency‐dependent natural selection maintaining these polymorphisms in time.

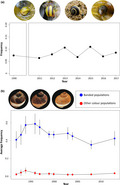

## 1 INTRODUCTION

The existence of color polymorphisms is widespread across taxa and has been widely studied (Cuthill et al., 2017; Hugall & Stuart‐Fox, 2012; McKinnon & Pierotti, 2010; Svensson, 2017). Color usually has a relatively simple genetic inheritance, and genotypes can be directly inferred from the phenotype. Moreover, it often impacts fitness (Cordero, 1990; Olsson et al., 2013; Schmutz & Berryere, 2007). The maintenance of such polymorphisms is not trivial as directional natural selection and genetic drift typically tend to erode diversity over time (Lewontin, 1974; Nielsen, 2005). However, in certain conditions both selection and genetic drift can maintain a polymorphism for generations (Hartl & Clark, 2014). For example, genetic diversity can be maintained by a balance between migration and mutation and genetic drift, in particular, when the effective population size is sufficiently high and the trait's effect on fitness is either weak or absent (e.g., Cook, 1992; Hoffman et al., 2006; Oxford, 2005). Therefore, for years, there was a debate on whether polymorphic traits were the result of stochastic or deterministic processes (Hartl & Clark, 2014; Kimura, 1991; Wright, 1977).

One of the most powerful and intuitive deterministic mechanisms by which polymorphisms can be maintained in natural populations is negative frequency‐dependent selection (NFDS; Ayala & Campbell, 1974; Fitzpatrick et al., 2007; Svensson & Connallon, 2019; Trotter & Spencer, 2008). Additional mechanisms are spatially or temporally heterogeneous selection (or multiple niche selection model; Levene, 1953) and overdominance. Importantly, the different mechanisms explaining polymorphisms are not mutually exclusive but can occur simultaneously.

**FIGURE 1:**
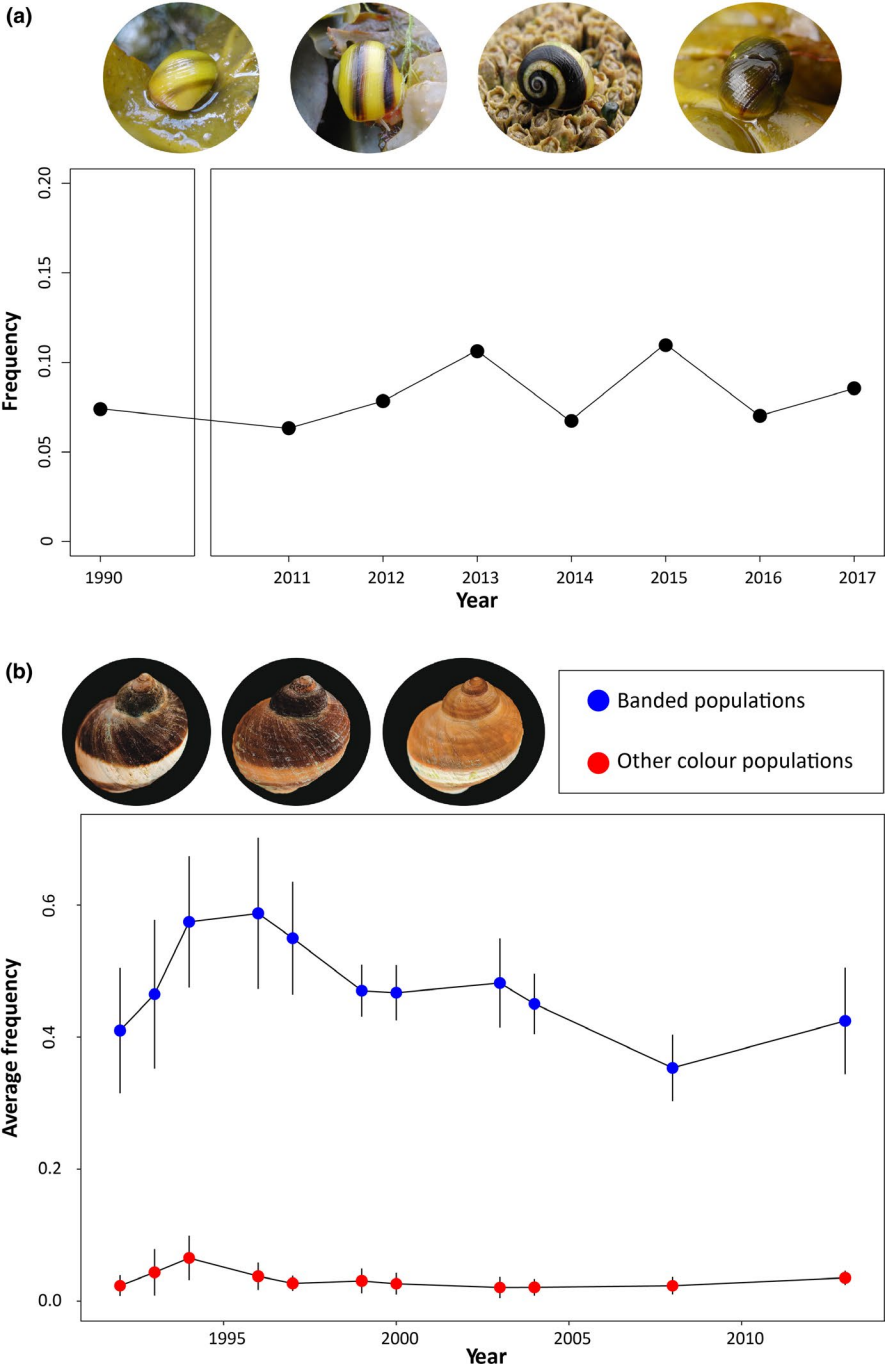
Banded individuals of *Littorina fabalis* and *Littorina saxatilis* and their frequency across years. (a) Examples of *L. fabalis* individuals and sample frequency over a period of 7 years in the Spanish population of Abelleira; photographs by Dr. Daniel Estévez. (b) Examples of *Littorina saxatilis* individuals and average frequency over a period of 21 years for Swedish populations recolonized with banded (blue) or white and/or red (red) individuals; photographs by Fredrik Pleijel

The banding polymorphism of the marine flat periwinkle *Littorina fabalis* (Turton, 1825) and the rough periwinkle *Littorina saxatilis* is ideal to study these matters as it tends to comply with most of the characteristics cited in the first paragraph: It has been shown to have a simple genetic basis (Ekendahl & Johannesson, 1997; Kozminsky, 2011); it is pervasive (Figure 1; Johannesson & Butlin, 2017); and it is easily scored (Figure 1). Furthermore, these two species are good models for evolutionary research (reviewed in Rolán‐Alvarez et al., 2015). Both *L. fabalis* and *L. saxatilis* are intertidal rocky shore snails present on North Atlantic shores. They are gonochoric and polygamous and show internal fertilization. *Littorina fabalis* is an annual species; that is, overlap between generations is minor (Reid, 1996; Rolán‐Alvarez et al., 2015; Williams, 1992), while for *L. saxatilis,* we follow the assumption by Johannesson and Butlin (2017) that the Swedish wave ecotype of this species (from which the data of this study are derived) is reasonably annual as well. Accordingly, the effects of the different evolutionary forces can be estimated year by year. *L. fabalis* is oviparous (laying egg masses from which juvenile snails hatch) and associated with brown algae, for example, *Fucus* spp., while *L. saxatilis* is ovoviviparous (females carry the offspring during the larval development and give birth to miniature snails) and can be mostly found on rock surfaces. The two species have low dispersal ability, and migrations among localities separated by a few km are occasional, presumably by rafting (Johannesson & Butlin, 2017; Johannesson & Johannesson, 1995; Reid, 1996).

The data for this study were obtained from two independent research efforts. For *L. fabalis*, phenotypic data on banding frequency were obtained from a study on shell color and mate choice (Estévez et al., 2020), although the data on banding are presented here for the first time. For *L. saxatilis*, we used published data from a study by Johannesson and Butlin (2017; https://doi.org/10.5061/dryad.427p0). For estimation of genetic drift in *L. fabalis*, we used an estimation of effective population size performed from microsatellite data, whereas for *L*. saxatilis, we followed the same interpretation as previous authors. As frequency was captured along a time series for both datasets, we could estimate candidate evolutionary forces affecting the banding pattern polymorphism. In particular, we aimed at following a similar methodology to that shown by Martínez‐Rodríguez et al. (2019). Thus, the objectives of this study were as follows: (a) to determine the relative contribution of genetic drift in the maintenance of the banding polymorphism in *L. fabalis* and (b) to estimate whether a mechanism of NFDS contributes to the polymorphism in both species.

## 2 MATERIALS AND METHODS

### 2.1 Original sampling

Mating pairs and unmated individuals of *L. fabalis* were collected from the *Fucus* spp. canopy in a rocky shore located in Abelleira, A Coruña, NW Spain (42°48′0.30″N– 9°1′14.87″W), for a period of 7 years from 2011 to 2017 (see further details in Estévez et al., 2020). The density of the population is relatively large compared with other populations, with tens of individuals per algae. All individuals were collected at the lower part of the intertidal to avoid collecting individuals of the sibling species *L. obtusata*, which inhabits the upper part (Williams, 1990). Additionally, the inspection of the sexual anatomy (see Reid, 1990, 1996) and a molecular analysis using species‐specific microsatellite loci (see Carvalho et al., 2016), performed with the 2014 sample, indicated that *L. obtusata* individuals accounted only for 1.4% of juveniles and 1.0% of females, both difficult to classify. This indicates that the amount of error incorporated due to misidentified individuals was negligible. Identified individuals of *L. obtusata* were discarded before the analyses. The presence/absence of bands was recorded for a total of 4,717 specimens across the 7 years. Shell band frequencies in this population were never artificially manipulated.

Frequencies of banded *L. saxatilis* individuals from eight Swedish skerries were obtained by Johannesson and Butlin (2017) from a sample of 11 years out of a period of 21 years to evaluate the contribution of different evolutionary forces to the maintenance of rare color morphs. The skerries were recolonized with individuals of a single color morph coming all from a larger island (58°49′18.6″N; 11°02′19.2″E), after a toxic algal bloom wiped out the original populations in 1992 (Johannesson & Johannesson, 1995). A group of skerries (W1, W2, W3, and R) were recolonized with white and red snails, while another group of skerries (B1, B2, and B3) were recolonized with banded snails at the start of the experiment (Johannesson & Butlin, 2017). In subsequent samplings, there was no manipulation of morph frequencies. Notice that similar results in both kinds of populations would suggest that manipulation itself had no effect in our fitness estimate. One of the skerries did not have enough data to evaluate the NFDS pattern and consequently was removed from the analysis (W4). Original results from Johannesson and Butlin (2017) pointed out that either NFDS or overdominance was possible mechanisms for the persistence of low frequencies of banded snails in most populations of the area.

### 2.2 Estimation of genetic drift

The effective population size (*N*_e_) obtained by Estévez et al. (2020) was used here to test whether changes in allele frequencies for the polymorphism of bands in *L. fabalis* could be explained by genetic drift. The genotypes of the microsatellites analyzed in this study were used to estimate the *N*_e_ using the linkage disequilibrium method as implemented in the software NeEstimator v.2.01 (Do et al., 2014). We assumed a model of one locus and two alleles with banded dominant over unbanded pattern (following Ekendahl & Johannesson, 1997; Johannesson & Butlin, 2017; Kozminsky, 2011). Then, the observed changes in frequencies between years, assuming that each year represents a generation (see Williams, 1992), were compared with the expected values under a null model of genetic drift, where variance in the shift of allele frequency in one generation is given by(Fisher, 1922):

(1) $$\sigma_{q1}^{2}=\frac{p_{0}q_{0}}{2N_{e}}$$

where *p*_0_ and *q*_0_ are the allele frequencies in the previous year. The null hypothesis (i.e., frequency changes in a trait are caused by genetic drift) would be accepted in Abelleira if the observed values of allele frequencies in 1 year fell within the 95% confidence interval of the estimated changes in frequency from the previous year under the null model of genetic drift (for a particular *N*_e_ value). To test this, we used the mean estimate of *N*_e_ and its 95% confidence interval values.

Johnannesson and Butlin (2017) tested whether genetic drift alone could explain changes in frequency in rare colors comparing the observed regression between phenotypic frequencies in time intervals with a maximum‐likelihood model of genetic drift, dependent on effective population size and sampling error, with slope 1 and intercept 0 and under the Hardy–Weinberg equilibrium. However, they could not separate nor estimate these parameters (sampling error and effective population size); rather, they rejected that genetic drift alone could explain their observations.

### 2.3 Estimate of fitness

Fitness was estimated based on the frequency changes between 2 consecutive years (see, e.g., Cook et al., 1999; Martínez‐Rodríguez et al., 2019). This interannual fitness estimate represents the response to selection under a quantitative genetic approach, and assuming a Mendelian genetic basis with no environmental effects, the response is equivalent to the total selection differential (Walsh & Lynch, 2018). Fitness was estimated by the cross‐product estimator, which is the most suitable for qualitative traits (*W*; Knoppien, 1985, Rolán‐Alvarez & Caballero, 2000, Martínez‐Rodríguez et al., 2019) and which can capture long‐term fitness better than other methods (see Brommer et al., 2004). One of its major advantages is its frequency independence, which makes it very suitable for studying frequency‐dependent mechanisms (Martínez‐Rodríguez et al., 2019). The *W* estimator is calculated as.

(2) $$W=\frac{{\text{AS}}_{B}\times {\text{BS}}_{U}}{\text{BS}\times {\text{AS}}_{U}},$$

where AS and BS are the frequencies of individuals (B‐banded, U‐unbanded) after (+1 year) and before (+0 year) selection, respectively. Equation 2) is the calculation of fitness for banded individuals, while fitness for unbanded individuals is its inverse. This estimate is equivalent to *1‐s* in Cook et al. (1999) where *s* is the fitness estimate for what they called *method by ratios*. Significance of the *W* estimate was performed by bootstrapping observed trait frequencies, before (BS) and after (AS) selection independently (after 10,000 iterations) using the program JMATING V. 1.0.8 (Carvajal‐Rodríguez & Rolán‐Alvarez, 2006).

### 2.4 Analysis of deterministic balancing mechanism

A linear regression of the percentage of banded individuals on interannual fitness [expressed as the log_10_(*W*)] was carried out to find patterns of frequency‐dependent selection. A single linear regression ANOVA was used to assess the significance of the relationship between fitness and frequency in the *L. fabalis* and *L. saxatilis* populations across the sampling years.

In *L. saxatilis*, the same hypothesis, namely that fitness is a function of frequency of the allele for banding, was evaluated in each population. Complementarily, the same hypothesis was estimated using all populations by two different methods: (a) Using the average of *β*, the parametric value of the regression coefficient, from all populations, a *t* test was employed to evaluate the null hypothesis *β* = 0; and (b) Fisher's test was used for combining the probabilities, which takes the *p* value from each independent test and follows the equation:

(3) $$\chi^{2}=‐2\sum_{i=1}^{k}\ln p$$

where *k* is the number of different tests (Sokal and Rohlf, 1995). The significance of this test is evaluated against a chi‐square distribution with 2*k* degrees of freedom.

## 3 RESULTS

### 3.1 Evaluating the role of genetic drift

**FIGURE 2:**
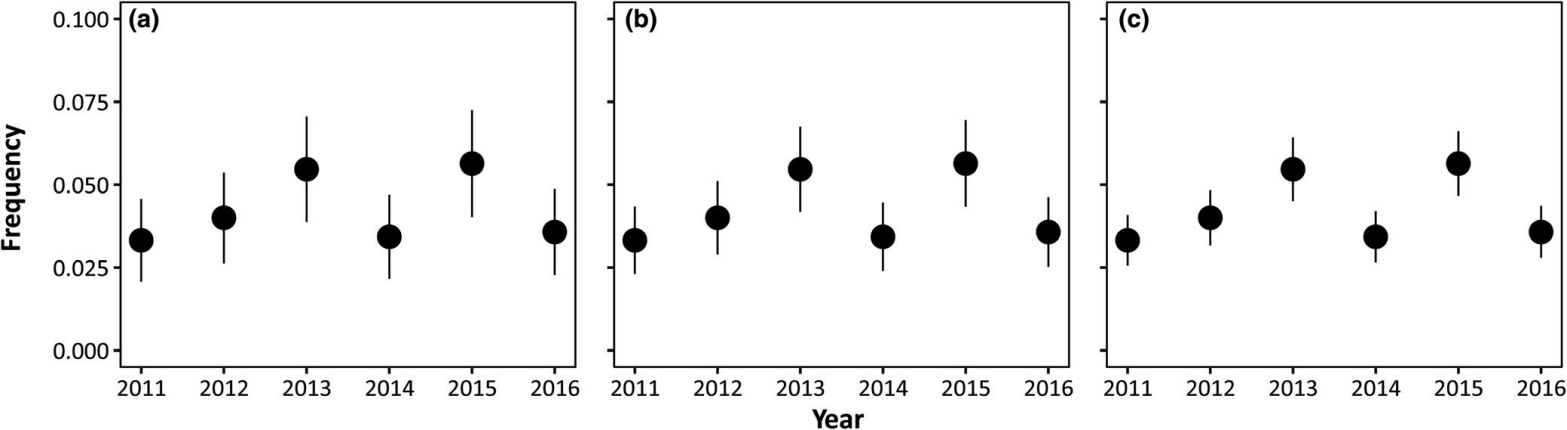
Expected changes in allelic frequency for the band polymorphism in *Littorina fabalis* for the Spanish population of Abelleira assuming genetic drift (Equation 1) in main text). Each dot represents the allelic frequency for the banded polymorphism observed in the year under the assumption of the Hardy–Weinberg equilibrium. The bars indicate the 95% confidence interval of the expected frequencies under genetic drift for the next year, using the minimum (a), mean (b), and maximum (c) estimated values of effective population size (394, 600, and 1,076, respectively)

The available *N*_e_ values (mean = 600; CI_95%_ = 394–1076) for *L. fabalis* from NW Spain were used to estimate the variance in the change in allele frequency for banding pattern (Equation 1), over one generation assuming only drift. Calculating the expected variance using the mean, minimum, and maximum values of *N*_e_ showed that the allele frequency changes between years could not be explained by drift alone. The range of changes from 2012 to 2015 was not observed to fall within the ranges predicted by a model of genetic drift alone using the mean or the maximum *N*_e_. Using the minimum *N*_e_, results varied only from 2012 to 2013 where the change in frequency could be explained by drift (Figure 2).

Genetic drift in *L. saxatilis*, investigated by Johannesson and Butlin (2017), was discarded as the explanatory force in most cases, although the contribution of genetic drift to the frequency change was not excluded in remaining models.

### 3.2 Estimates of fitness

#### 3.2.1 Littorina fabalis

Overall, the percentage of banded individuals never surpassed 12% in the Abelleira population (Table 1). Estimates of fitness were significant in three yearly intervals, 2013–2014, 2014–2015, and 2015–2016 (Table 1).

**TABLE 1:** Estimates of interannual (W ± *SD*) fitness for banded *Littorina fabalis* individuals using the cross‐product estimator (*W*) across a period of 7 years. The total number of individuals sampled (*N*) and the percentage of banded individuals correspond to the first year in each interval (2017: N, 916; % banded, 8.51). Notice that since the *W* estimator is a relative measurement of fitness, fitness for unbanded individuals is the inverse

Year interval	*N*	% banded	*W*
2011–2012	521	6.33	1.27 ± 0.19
2012–2013	708	7.91	1.38′ ± 0.15
2013–2014	574	10.63	0.61 ± 0.13
2014–2015	564	6.74	1.70 ± 0.13
2015–2016	593	10.96	0.61 ± 0.14
2016–2017	841	7.02	1.23 ± 0.15

**FIGURE 3:**
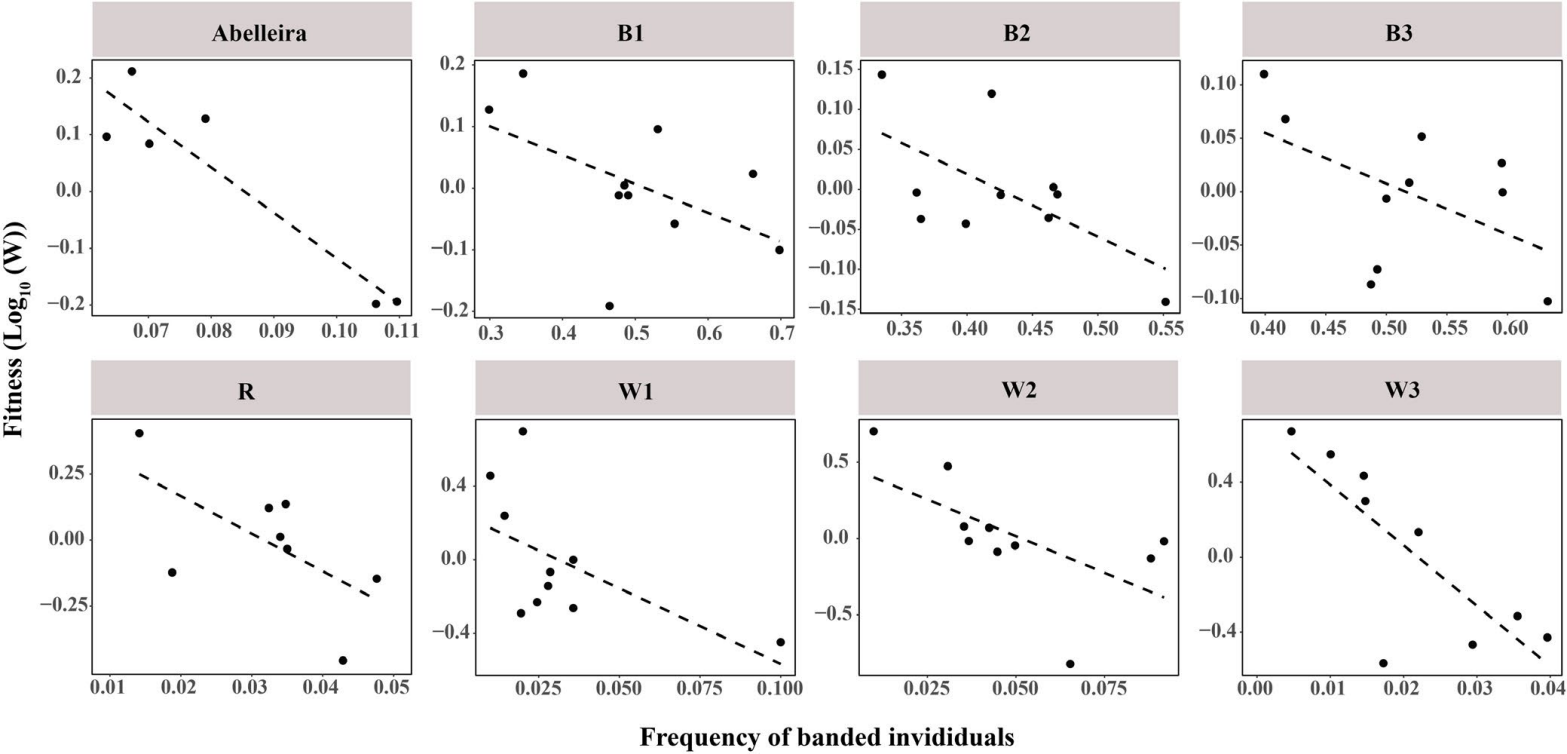
Observed negative relation between annual fitness (log 10 W) and frequency of banded *Littorina saxatilis* (B1, B2, B3, R, W1, W2, and W3) and *Littorina fabalis* (Abelleira) individuals in different sampled populations (notice that this frequency refers to the phenotype, not the allelic frequency). Notice that a random pattern does not necessarily produce such a trend as it may cause several changes in frequencies in the same direction (causing a direct association between fitness and frequency)

The linear model between frequency and fitness is represented in Figure 3. The regression line showed a negative relation between fitness and percentage of banded individuals (notice that both fitness and percentage are relative values, and hence, the same results will be observed for unbanded individuals). The relationship between frequency (using always the starting frequency, i.e., that of year + 0 in all intervals) and fitness was significant (*F*_1,4_ = 27.31, *p* =0.006).

#### 3.2.2 Littorina saxatilis

Fitness estimates for *L. saxatilis* in the seven populations (population W4 not included) are shown in Table 2. Significant estimates were observed in both decreases and increases in frequency across year intervals. These were particularly high in cases where frequency changes were high: Fitness increased much more from low starting frequencies and *vice versa*.

**TABLE 2:** Estimates of interannual (W ± *SD*) fitness for banded *Littorina saxatilis* individuals for populations manipulated for conspicuous (W1, W2, W3, and R) and banded (B1, B2, and B3) individuals. Notice that since the W estimator is a relative measurement of fitness, fitness for unbanded individuals is the inverse. NA = impossible to calculate

Year Interval	W1		W2		W3		R	
	% banded	W	% banded	W	% banded	W	% banded	W
1992–1993	2.79	0.71 ± 0.76	3.08	3.18** ± 0.17	0.00	NA	3.48	1.39 ± 0.44
1993–1994	2.01	5.42*** ± 0.16	9.18	0.96 ± 0.38	1.46	2.79* ± 0.31	4.76	0.70 ± 0.50
1994–1996	10.00	0.33 ± 0.55	8.81	0.72 ± 0.33	3.96	0.36^′^ ± 0.33	3.40	1.03 ± 0.81
1996–1997	3.57	1.00 ± NA	6.53	0.14*** ± 0.12	1.48	2.02 ± 0.60	3.50	0.92 ± 0.79
1997–1999	3.57	0.54 ± 0.50	0.99	5.26** ± 0.17	2.94	0.34 ± 0.45	3.24	1.34 ± 0.50
1999–2000	1.95	0.51 ± 0.77	4.98	0.90 ± 0.55	1.01	3.63* ± 0.29	4.29	0.34* ± 0.28
2000–2003	1.00	2.91 ± 0.53	4.48	0.81 ± 0.66	3.55	0.48 ± 0.52	1.50	NA
2003–2004	2.86	0.85 ± 1.07	3.67	0.96 ± 1.13	1.72	0.27 ± 0.41	0.00	NA
2004–2008	2.45	0.58 ± 0.76	3.54	1.21 ± 0.58	0.47	4.77* ± 0.32	1.88	0.75 ± 1.06
2008–2013	1.44	1.75 ± 0.71	4.25	1.19 ± 0.54	2.20	1.37 ± 0.75	1.42	2.59^′^ ± 0.37

Year Interval	B1		B2		B3	
	% banded	W	% banded	W	% banded	W
1992–1993	34.59	2.14*** ± 0.12	36.48	0.88 ± 0.20	51.89	1.04 ± 0.24
1993–1994	53.09	1.73* ± 0.18	33.49	1.73* ± 0.15	52.91	1.31 ± 0.15
1994–1996	66.18	1.18 ± 0.38	46.60	1.01 ± 0.26	59.60	1.00 ± 0.22
1996–1997	69.81	0.54* ± 0.22	46.91	0.97 ± 0.21	59.52	1.17 ± 0.19
1997–1999	55.41	0.76 ± 0.22	46.23	0.86 ± 0.18	63.32	0.58** ± 0.12
1999–2000	48.50	1.02 ± 0.20	42.57	0.97 ± 0.21	50.00	0.97 ± 0.20
2000–2003	49.02	0.95 ± 0.38	41.88	1.71* ± 0.16	49.25	0.74 ± 0.19
2003–2004	47.73	0.95 ± 0.35	55.17	0.54** ± 0.14	41.67	1.33 ± 0.19
2004–2008	46.46	0.49*** ± 0.10	39.91	0.85 ± 0.17	48.73	0.70* ± 0.15
2008–2013	29.91	1.57* ± 0.13	36.15	0.98 ± 0.21	39.90	1.59** ± 0.13

* *p* ≤0.05,

** *p* ≤ 0.01,

*** *p* ≤ 0.001, and

^′^ *p* = 0.05.

Johannesson and Butlin (2017) studied banded frequency in eight populations. Our reanalysis presented here shows the existence of a frequency‐dependent pattern, as fitness of banded specimens varies negatively with its frequency, and this occurred with the population being manipulated either for color or for banding at the start of the experiment (Table 2; Figure 3). Furthermore, notice that these patterns represent a period of 21 years in all populations, supporting the stability of the mechanism and its persistence in spite of the initial frequencies. The regression was significant only in one population although four more were marginally significant (Table 3). However, when the whole subset of populations is tested simultaneously, the result is significant: A *t* test on the average of *β* coefficients indicated an inverse relationship between fitness and frequency, being such average equal to −0.61; additionally, Fisher´s test combining the probabilities of the seven ANOVAs was strongly significant (Table 3). Following these two results, the observed pattern adjusts to what it would be expected under NFDS (Figure 3).

**TABLE 3:** Linear regression reanalysis of fitness (log 10 W, dependent variable) over the percentage of banded individuals (predictor) in the 8 populations studied by Johannesson and Butlin (2017). One of the populations (3) was not included because of the absence of banded individuals per year. Two tests for the combined information of the 7 populations are provided (see M&M): a *t* test for the *β* mean and a Fisher test (chi‐squared test) for combining probabilities from independent tests in the column probability

Population	Banded Frequency	*N*	*R* ^2^	Beta	*F* test	Probability
W1	0.05 ± 0.026	10	0.38	−0.62	4.97	0.056
W2	0.03 ± 0.025	10	0.34	−0.58	4.13	0.077
W3	0.02 ± 0.012	9	0.62	−0.79	11.66	0.011
R	0.43 ± 0.065	10	0.40	−0.63	5.27	0.051
B1	0.50 ± 0.122	10	0.27	−0.52	2.96	0.124
B2	0.52 ± 0.076	10	0.27	−0.52	2.90	0.127
B3	0.03 ± 0.011	8	0.40	−0.63	4.02	0.092
Combined test			−0.61*** ± 0.092			38.94***

Note

*N* is the number of points (between consecutive periods) in the regression. *R* ^2^ is the percentage of variance explained in the dependent variable by the predictor variable. Beta is the standardized regression coefficient in the regression. The *F* test and probability are corresponding values in the regression ANOVA.

*** *p* ≤.001.

## 4 DISCUSSION

The maintenance of genetic polymorphisms in the absence of balancing selection (i.e., under mutation–drift–migration equilibrium) has been documented in a number of cases, with examples including color polymorphisms in the leopard frog *Rana pipiens* (Hoffman et al., 2006) and in the periwinkle *Littoraria pallescens* (Cook, 1992; Parsonage & Hughes, 2002). Even polymorphisms in *Cepaea* snails, where there is a long history of research on selective mechanisms, have been attributed to some extent to equilibriums between migration and drift (Bellido et al., 2002). Some of these species have large population sizes, and consequently, neutral processes could be associated with the transitory changes of these genetic polymorphisms in the absence of selective forces. Contrarily, genetic drift is expected to reduce genetic diversity in *L. fabalis* at Abelleira as the species presumably suffers strong bottlenecks during winter (Rolán‐Alvarez et al., 1995; Williams, 1996; although other populations outside NW Spain can be more dense and stable; see Reid, 1996). The average percentage of banded individuals in the *L. fabalis* population, Abelleira, was 8.3% ± 1.9 *SD*. These observations suggest that although genetic drift could explain subtle changes in frequency for the polymorphism (Figure 2), when a change occurs over a certain threshold, a balancing mechanism protects the polymorphism from being lost. The evidence in the related species, *L. saxatilis*, is similar. On the one hand, it is a low‐dispersal species that shows intermediate density populations along the rocky shore and is expected to have low effective population size in certain areas (Fernández et al., 2005, but see Reid, 1996). On the other hand, genetic diversity in *L. saxatilis* can rapidly recover after strong bottlenecks, probably because one single female is able to recolonize one population having a brood pouch with hundreds of embryos from up to 23 different sires (Panova et al., 2010; Rafajlović et al., 2013). The averaged percentage of banded individuals across time in the studied populations of *L. saxatilis* varied greatly, with the skerries manipulated for banded individuals showing the greatest percentages (B1—50.1% ± 12.3 *SD*; B2—42.5% ± 12.3 *SD*; B3—51.7 ± 7.6 *SD*) and the ones manipulated for other colors showing the lowest (W1—3.2% ± 2.5 *SD*; W2—4.9% ± 2.6 *SD*; W3—1.9% ± 1.3 *SD*; R—2.8% ± 1.5 *SD*). The banded shell pattern in this species persisted over time, and drift could not account for all observed changes in banding frequency (Johannesson & Butlin, 2017). These trends in both species are similar to what has been proposed in candy‐striped spider (Oxford, 2005). In this example, color polymorphism was found to be almost neutral within a specific range of frequencies with selection regimes being very weak. However, when drift pushed frequency of a color morph over this range, evidence was found that some form of natural selection pushed back the frequency to previous values.

Interannual fitness of banded individuals was found to decrease with frequency, thus fulfilling the necessary condition for a mechanism of NFDS (for a similar approach, see Cook et al., 1999; Martínez‐Rodríguez et al., 2019). This was observed in both species (Figure 3; Table 2) and independently of the initial or average frequency of the phenotype. It could be argued that the initial frequencies obtained for *L. saxatilis* could have a profound effect on the evolutionary outcome, especially whether drift had a significant contribution. However, in the period of 21 years, a balancing mechanism was observed for populations either with low or higher frequencies (Figure 3). Johannesson and Butlin (2017) were supportive of a mechanism of NFDS maintaining rare polymorphisms in *L. saxatilis* but suggested that overdominance could also be a candidate mechanism. However, although overdominance has been observed to maintain polymorphisms in a number of cases (Kellenberger et al., 2019; Schou et al., 2017), it is somewhat unlikely to produce the patterns observed in Figure 3. On the contrary, one argument supporting overdominance would be that both types of experimental populations in *L. saxatilis* (manipulated for banding or not) would maintain the observed equilibrium frequencies irrespectively of the initial frequencies. Notice that our observations cannot definitively support or reject overdominance, since it could produce a trend similar to a frequency‐dependent pattern, at least for a limited number of generations. Importantly, associative overdominance where the polymorphism is controlled by a supergene or a chromosomal inversion is gaining momentum in explaining adaptation as more of these regions are being found in various taxa (Wellenreuther & Bernatchez, 2018). In fact, several chromosomal inversions have been found in *L. saxatilis* (Faria et al., 2019). Deleterious mutations can accumulate in haplotypes containing inversions causing homozygotes to have lower fitness. Such haplotypes could be maintained in heterozygotes, which would be in low frequency (see Jamie & Meier, 2020, and references therein). Thus, this mechanism cannot be rejected given our current observations and deserves future consideration.

Another possible balancing mechanism is heterogeneous selection, which could theoretically maintain a trend similar to that observed in Figure 1 for *L. fabalis*. However, fitness in this case would be dependent on the frequency of exploitable habitats rather than other polymorphism variants’ frequencies, and thus, more erratic variation than that observed in Figure 1 would be expected. Such erratic variation would indeed be expected in *L. fabalis* due to the relative complexity of its habitat: The brown algae canopy presents yellow, brown, and green color variations, and its density fluctuates along the year with strong reductions during winter (Rolán‐Alvarez et al., 1995). On the other hand, *L. saxatilis* from the studied dataset inhabited a relatively simple rocky shore that remains identical over the year. Overall, we follow the same argument exposed by Brisson (2018) that any particular rare variant would not increase its fitness unless its most suitable background is encountered frequently. Therefore, the most plausible interpretation is that there is a true frequency‐dependent natural selection mechanism involved although we cannot neither prove nor reject heterogeneous selection based on our observations.

Frequency‐dependent, predator‐driven selection (apostatic selection) has been suggested for the band polymorphism in the closely related species *L. obtusata* (Smith, 1976). This kind of selection has also been proposed in guppies (Olendorf et al., 2006), salamanders (Fitzpatrick et al., 2009), and land snails (Tucker, 1991), among others. Most of these studies convey in hypothesizing that apostatic selection is a result of search image employment by predators, which adjusts to the frequency of the different colors or patterns (Bond, 2007). This in turn would imply that the polymorphism evolves in a “fine‐grained” environment, that is, homogeneous environments where all polymorphic variants are equally cryptic (Surmacki et al., 2013). The main predator of both *L. fabalis* and *L. saxatilis* is the shore crab *Carcinus maenas* (Ekendahl, 1998; Williams, 1992); however, given the poor vision of crabs, intertidal fish or birds seem to be better candidates for visual predation (see Johannesson & Ekendahl, 2002; Reimchen, 1989). A possible combination of both apostatic selection and heterogeneous selection could occur in the sense that the environmental complexity could determine the strength of apostatic selection and crypsis (Bond & Kamil, 2006).

The above interpretation assumes that viability should be the main component of fitness involved. However, since we are taking an integrative estimation of fitness, we can only speculate about the major causes for such pattern. Otherwise, we can say that sexual selection is not contributing to the maintenance of the band polymorphism. The way data were obtained for *L. fabalis* in Estévez et al. (2020) allowed us to estimate this component of natural selection, and we found nor a pattern of NFDS using sexual fitness (females: *F*_1,7_ = 0.53, *p* =0.497| males: *F*_1,7_ = 0.66, *p* = 0.452) nor assortative mating (*I_PSI_* = 0.04; *SD* = 0.12, *p* = 0.7818). We believe therefore that future studies should focus on the viability fitness components and designing predatory experiments to disentangle the biological mechanism in detail both in the field and in the laboratory.

In summary, we have found that banded pattern in *L. fabalis* and *L. saxatilis* shows a NFDS pattern of change in polymorphism frequencies along years. As several other intertidal gastropods show variation in shell banding (such as *Nucella lapillus* and *L. obtusata*), we believe that similar explanations could be invoked in the evolution of this polymorphism, especially on those showing direct development, and consequently being more prone to local adaptation. For example, a common predator or group of predators could be driving local adaptation in coexisting snail species of similar sizes causing apostatic selection. Further investigations on the ultimate biological mechanism causing this pattern will be needed, as well as conclusive work that can elucidate the relative role of either apostatic or heterogeneous selection.

## CONFLICT OF INTEREST

We hereby declare that no author of the research presented here has any conflict of interest.

## AUTHOR CONTRIBUTIONS

Daniel Estévez: Conceptualization (equal); Data curation (lead); Formal analysis (lead); Investigation (equal); Methodology (equal); Software (lead); Visualization (lead); Writing‐original draft (lead); Writing‐review & editing (equal). Juan Galindo: Conceptualization (supporting); Formal analysis (supporting); Investigation (supporting); Methodology (equal); Supervision (equal); Writing‐review & editing (equal). Emilio Rolán‐Alvarez: Conceptualization (lead); Data curation (supporting); Formal analysis (equal); Funding acquisition (lead); Investigation (equal); Methodology (equal); Project administration (equal); Supervision (lead); Visualization (supporting); Writing‐review & editing (equal).

## Data Availability

Information on frequencies of banded individuals across temporal and spatial scale, and estimates of fitness can be found in Dryad, https://doi.org/10.5061/dryad.2280gb5rb.
